# Association between adipocyte fatty acid-binding protein with left ventricular remodelling and diastolic function in type 2 diabetes: a prospective echocardiography study

**DOI:** 10.1186/s12933-020-01167-5

**Published:** 2020-11-24

**Authors:** Mei-Zhen Wu, Chi-Ho Lee, Yan Chen, Shuk-Yin Yu, Yu-Juan Yu, Qing-Wen Ren, Ho-Yi Carol Fong, Pui-Fai Wong, Hung-Fat Tse, Siu-Ling Karen Lam, Kai-Hang Yiu

**Affiliations:** 1 Division of Cardiology, Department of Medicine, the University of Hong Kong Shenzhen Hospital, Shen Zhen, China; 2 Division of Cardiology, Department of Medicine, the University of Hong Kong, Room 1929C, Block K, Queen Mary Hospital, Hong Kong, China; 3 Division of Endocrinology, Department of Medicine, the University of Hong Kong, Queen Mary Hospital, Hong Kong, China; 4 Department of Ultrasound, Shenzhen Hospital, Southern Medical University, Shen Zhen, China

**Keywords:** AFABP, Echocardiography, Major adverse cardiovascular events, Type 2 diabetes

## Abstract

**Background:**

The relationship between adipocyte fatty acid-binding protein (AFABP) and cardiac remodelling has been reported in cross-sectional studies, although with conflicting results. Type 2 diabetes mellitus (T2DM) is associated with left ventricular (LV) hypertrophy and diastolic dysfunction, as well as elevated circulating AFABP levels. Here we investigated prospectively the association between AFABP with the longitudinal changes of cardiac remodelling and diastolic dysfunction in T2DM.

**Methods:**

Circulating AFABP levels were measured in 176 T2DM patients without cardiovascular diseases (CVD) at baseline. All participants received detailed transthoracic echocardiography both at baseline and after 1 year. Multivariable linear and Cox regression analyses were used to evaluate the associations of circulating AFABP levels with changes in echocardiography parameters and incident major adverse cardiovascular events (MACE), respectively.

**Results:**

The median duration between baseline and follow-up echocardiography assessments was 28 months. Higher sex-specific AFABP quartiles at baseline were associated with increase in LV mass and worsening of average E/e′ (all P < 0.01). Multivariable linear regression demonstrated that AFABP in the highest quartile was independently associated with both increase in LV mass (β = 0.89, P < 0.01) and worsening of average E/e′ (β = 0.57, P < 0.05). Moreover, multivariable Cox regression analysis showed that elevated baseline circulating AFABP level independently predicted incident MACE (HR 2.65, 95% CI 1.16–6.05, P < 0.05) after adjustments for age, sex, body mass index, glycated haemoglobin, hypertension, dyslipidemia and presence of chronic kidney disease.

**Conclusion:**

Circulating AFABP level at baseline predicted the development of LV hypertrophy, diastolic dysfunction and MACE in T2DM patients without CVD.

## Background

Type 2 diabetes mellitus (T2DM) is associated with left ventricular (LV) hypertrophy and diastolic dysfunction independent of underlying coronary artery disease (CAD) [1–3], and contributes to the development of major adverse cardiovascular events (MACE) [4]. With rising global prevalence of T2DM, a deeper understanding of the mechanisms in diabetes related LV remodelling and myocardial dysfunction is thus of paramount importance.

Fatty acid-binding protein has been linked with cardiovascular diseases [5, 6]. Adipocyte fatty acid-binding protein (AFABP) is a lipid chaperone protein mainly expressed in adipocytes and macrophages. It is also secreted by adipocytes and circulates in the bloodstream [7]. High circulating AFABP level was found in obese individuals [7] and predicted the development of metabolic syndrome [8], gestational diabetes [9], T2DM [10], carotid atherosclerosis [11] and cardiovascular diseases (CVD) [12]. Raised circulating AFABP level has also been shown to associate with MACE in patients with CAD [13], as well as aortic arterial stiffness and cardiovascular mortality in patients with T2DM [14–16].

In heart failure, AFABP positively correlated with N-terminal pro-brain natriuretic peptide (NT-proBNP) and clinical severity [17]. In a prospective study, high AFABP level also predicted incident heart failure in older adults aged ≥ 65 years [18]. However, the relationship between AFABP and LV remodelling is less straight forward, and is limited by evidence mostly derived from cross-sectional studies. AFABP has been associated with diastolic dysfunction in the general population [19], as well as in morbidly obese individuals [20]. On the other hand, while previous studies demonstrated that elevated AFABP level was associated with increased LV mass in overweight and obese individuals [21], two recent studies reported an inverse relationship [22, 23]. Moreover, although it is increasingly recognized that LV hypertrophy and diastolic dysfunction is common in T2DM patients, the relationship between AFABP and LV remodelling in T2DM remains to be elucidated. Therefore, we performed this prospective study to investigate the associations of circulating AFABP level with longitudinal changes of LV remodelling and diastolic dysfunction in patients with T2DM. We also evaluated the prognostic significance of AFABP in T2DM patients who did not have CVD at baseline.

## Methods

### Study population

All participants were recruited from the specialist medical clinics of Queen Mary Hospital, Hong Kong from July 2011 to August 2014. Participants who had T2DM but without CVD and severe structural heart disease were enrolled for baseline blood tests and echocardiography. Subsequently, they were invited for follow-up echocardiography after 1 year. The study was approved by the ethics committee of the West Cluster Hospital Authority of Hong Kong and all participants gave written informed consent prior to any study-related procedures. This study was part of the Chinese Diabetic Heart Study (CDATS) to evaluate the cardiovascular manifestations in Chinese T2DM patients, the pathophysiology behind and related potential therapies [24].

### Clinical and biochemical data

Clinical measurements and blood sampling were obtained on the same day after an overnight fast for at least 8 h. Detailed medical and medication histories including smoking status, hypertension, dyslipidemia, use of anti-hypertensive and anti-diabetic medications were documented. Anthropometric parameters including body weight and height were measured, with body mass index (BMI) calculated in kg/m^2^. Blood pressure was measured at the end of echocardiography examination after a 5-min rest. Fasting blood samples were obtained for measurements of glycated haemoglobin (HbA1c), glucose, lipid profile, serum creatinine (SCr) and stored in aliquots at -80 °C for further assay [25]. Estimated glomerular filtration rate (eGFR) was estimated according to the Chronic Kidney Disease Epidemiology Collaboration (CKD-EPI) Equation [26]. Serum AFABP level was measured by a sandwich Elisa Kit (Antibody and Immunoassay Services, University of Hong Kong) as described in previous studies [27, 28]. The intra- and inter-assay coefficients of variation in this kit were < 4.1% and < 4.5%, respectively, with a sensitivity limit of 0.39 ng/mL [28].

### Echocardiography measurement

Detailed transthoracic echocardiography examination was performed in all participants with commercially available echocardiography machines (Vingmed Vivid 7 or E9, General Electric Vingmed Ultrasound, Milwaukee, WI, USA) by two cardiologists at baseline and follow-up. Images were obtained using a 3.5-MHz transducer and digitally stored into 3 cardiac cycles for offline analysis by EchoPAC version 108.1.5 (General Electric Vingmed, Horten, Norway). 20 participants were randomly selected to assess the intra- and inter-observer reproducibility in the measurements of echocardiography parameters, estimated by Bland–Altman and intra-class correlation coefficients. The agreement of intraobserver and interobserver variability was accepted (Additional file 1: Table S1).

Inter-ventricular septal dimension at end-diastole (IVSd) and LV posterior wall thickness at end-diastole (LVPWd) were measured using two-dimensional echocardiography guided M-mode approach based on current recommendations [29]. LV mass was calculated according to the Devereux formula [30]. LV volumes and LV ejection fraction (LVEF) were measured using modified biplane Simpson’s method in apical four- and two-chamber views. Left atrial volume (LAV) was assessed by single-plane disk summation method in apical four-chamber view. LAV index (LAVi) was determined by LAV divided by body surface area. Pulsed-wave Doppler and tissue Doppler imaging were applied to assess LV diastolic function in apical four-chamber view. Peak transmitral flow velocity in early (E-wave) and late diastole (A-wave) was measured, E/A ratio was calculated. Peak velocity of septal and lateral mitral annulus in early diastole (e′) was measured by tissue Doppler imaging, and average E/e′ was calculated [31].

### Definition of variables and outcomes

In this study, the diagnosis of T2DM was based on criteria set out by the American Diabetes Association [32]. Hypertension was defined as BP ≥ 140/90 mmHg or the use of anti-hypertensive medications. Dyslipidemia was defined as fasting triglyceride (TG) ≥ 1.69 mmol/L, high-density lipoprotein cholesterol (HDL-C) < 1.04 mmol/L in men and < 1.29 mmol/L in women, low-density lipoprotein cholesterol (LDL-C) ≥ 2.6 mmol/L, or the use of lipid-lowering medications. CKD was defined as eGFR < 60 mL/min/1.73 m^2^.

MACE was defined as a composite of cardiovascular death, hospitalization for heart failure, non-fatal myocardial infarction and stroke developed as of 31^st^ March 2020. The follow up of MACE started after the baseline visit of all patients. All clinical outcomes were retrieved from the electronic clinical management system of the Hospital Authority, Hong Kong, where all hospitalization and causes of mortality for each individual were documented in detail.

### Statistical analysis

In this study, categorical variables were presented as frequencies (proportions), where continuous variables with normal distribution were expressed as mean ± standard deviation. Skewed variables such as AFABP, TG and eGFR were presented as median (interquartile range), and were logarithmically transformed to obtain near normality before analysis. Since sexual dimorphism was known to be present in serum AFABP levels, patients were divided into four groups based on sex-specific AFABP quartiles as previously described [28], with each quartile consisting of an equal number of men and women. The differences of variables across quartiles was performed by one-way analysis of variance and post hoc analysis by Bonferroni. Chi-square test or Fisher’s exact test was used as appropriate to compare the differences between categorical variables. Comparison of clinical characteristics or echocardiography parameters at baseline and follow-up were made by paired t test or McNemar test as appropriate. Kaplan–Meier survival curve with log-rank test was performed to compare MACE by AFABP quartiles. Multivariable linear regression was applied to examine the association between serum AFABP levels and the changes in echocardiography parameters. Multivariable Cox regression was performed to evaluate the association between AFABP and MACE. Variables that were statistically significant in univariate analyses or biologically relevant were included in the multivariable regression models. All statistical analyses were conducted using IBM SPSS 26.0 (https://www.IBM.com/SPSS), and a 2-sided P < 0.05 was considered statistically significant.

## Results

A total of 265 T2DM patients were recruited in this study. After excluding those with history of severe valvular heart disease (n = 3), CAD (n = 16), heart failure (n = 3), stroke (n = 6), atrial fibrillation (n = 2), malignancy (n = 4) and poor echocardiographic images (n = 10) at baseline, 221 participants were enrolled for further evaluation with blood tests and invited for follow-up echocardiography. Participants who did not have follow-up echocardiography included those who developed new-onset heart failure (n = 2), severe valvular heart disease (n = 1), stroke (n = 1), CAD (n = 1), end-stage renal failure (n = 2), malignancy (n = 4), death (n = 3), lost to follow-up (n = 16), or declined the invitation (n = 15) after their baseline visit. Therefore, 176 participants returned for follow-up echocardiography after a median interval of 28 months, and were included in the analysis of this study.

Baseline clinical characteristics of these 176 participants are shown in Table 1. The mean age of the participants was 60 ± 10 years and 53% of them were men. Their mean BMI was 26 ± 5 kg/m^2^ and their mean duration of diabetes was 17 ± 7 years. The prevalence of hypertension, dyslipidemia and CKD were 75.6%, 68.8% and 14.2%, respectively. Their median circulating AFABP levels at baseline were 29.59 (20.62–52.60) ng/mL in women and 19.22 (12.49–29.24) ng/mL in men. Participants with increasing AFABP quartile were significantly older, and had higher BMI and prevalence of hypertension, serum TG levels, and lower eGFR and HDL-C levels (all P < 0.01).

**Table 1:** Clinical characteristics of study participants at baseline stratified by serum AFABP quartiles

	Total (n = 176)	1^st^ Quartile (n = 43)	2^nd^ Quartile (n = 45)	3^rd^ Quartile (n = 44)	4^th^ Quartile (n = 44)	P value
Women AFABP level (ng/mL)	29.59 (20.62–52.60)	< 20.62	20.62–29.58	29.59–52.60	> 52.60	
Men AFABP level (ng/mL)	19.22 (12.49–29.24)	< 12.49	12.49–19.21	19.22–29.24	> 29.24	
Clinical characteristics						
Age (years)	60 ± 10	57 ± 8	59 ± 10	59 ± 9	65 ± 9^†#&^	* < 0.01*
Men, n (%)	94 (53.4)	23 (53.5)	24 (53.3)	23 (52.3)	24 (54.5)	-
Diabetes duration (years)	17 ± 7	18 ± 7	16 ± 8	18 ± 8	15 ± 7	0.19
BMI (kg/m^2^)	26 ± 5	23 ± 4	26 ± 4^¶^	27 ± 5^§*^	28 ± 4^†#&^	* < 0.01*
SBP (mmHg)	137 ± 18	130 ± 19	140 ± 20	136 ± 16	140 ± 18	* < 0.05*
DBP (mmHg)	80 ± 9	77 ± 8	81 ± 9	82 ± 9^§^	78 ± 9	* < 0.05*
Smoker, n (%)	47 (26.7)	9 (20.9)	9 (20.0)	16 (36.4)	13 (29.5)	0.26
Medical history						
Hypertension, n (%)	133 (75.6)	25 (58.1)	34 (75.6)	35 (79.5)	39 (88.6)	* < 0.01*
Dyslipidemia, n (%)	121 (68.8)	26 (60.5)	30 (66.7)	31 (70.5)	34 (77.3)	0.39
CKD, n (%)	25 (14.2)	2 (4.7)	3 (6.7)	4 (9.1)	16 (36.4)	* < 0.01*
Blood chemistry						
HbA1c (%)	7.68 ± 1.26	7.70 ± 1.23	7.75 ± 1.45	7.53 ± 0.99	7.73 ± 1.36	0.83
Fasting glucose (mmol/L)	8.16 ± 2.73	8.07 ± 2.45	8.50 ± 2.91	7.79 ± 2.10	8.26 ± 3.33	0.66
eGFR^a^ (ml/min/1.73m^2^)	88.69 (73.35–98.23)	93.22 (86.74–100.09)	93.03 (83.72–101.29)	91.53 (69.31–98.50)	70.95 (53.66–84.99)^†#&^	* < 0.01*
Total cholesterol (mmol/L)	4.31 ± 0.81	4.31 ± 0.88	4.29 ± 0.71	4.39 ± 0.89	4.22 ± 0.75	0.80
HDL-C (mmol/L)	1.30 ± 0.35	1.42 ± 0.42	1.37 ± 0.31	1.28 ± 0.35	1.14 ± 0.22^†#^	* < 0.01*
LDL-C (mmol/L)	2.38 ± 0.63	2.26 ± 0.57	2.43 ± 0.59	2.57 ± 0.72	2.26 ± 0.60	0.07
Triglyceride^a^ (mmol/L)	1.20 (0.80–1.70)	0.90 (0.70–1.40)	1.00 (0.70–1.35)	1.60 (1.13–2.10)^§*^	1.40 (1.00–1.90)^†#^	* < 0.01*
Medications						
Insulin, n (%)	79 (44.9)	19 (44.2)	19 (42.2)	20 (45.5)	21 (47.7)	0.96
Metformin, n (%)	166 (94.3)	40 (93.0)	43 (95.6)	43 (97.7)	40 (90.9)	0.45
Sulfonylureas, n (%)	93 (52.8)	27 (62.8)	23 (51.1)	21 (47.7)	22 (50.0)	0.50
Gliptins, n (%)	36 (20.5)	13 (30.2)	6 (13.3)	6 (13.6)	11 (25.0)	0.13
ACEI/ARB, n (%)	107 (60.8)	20 (46.5)	28 (62.2)	28 (63.6)	31 (70.5)	0.13
β-Blocker, n (%)	60 (34.1)	10 (23.3)	14 (31.1)	14 (31.8)	22 (50.0)	0.06
CCB, n (%)	83 (47.2)	12 (27.9)	19 (42.2)	26 (59.1)	26 (59.1)	* < 0.01*
Diuretics, n (%)	16 (9.1)	–	5 (11.1)	4 (9.1)	7 (15.9)	0.07
Statin, n (%)	94 (53.4)	22 (51.2)	24 (53.3)	20 (45.5)	28 (63.6)	0.38

Hypertension was defined as BP ≥ 140/90 mmHg or the use of anti-hypertensive medications. Dyslipidemia was defined as fasting triglyceride ≥ 1.69 mmol/L, high-density lipoprotein cholesterol < 1.04 mmol/L in men and < 1.29 mmol/L in women, low-density lipoprotein cholesterol ≥ 2.6 mmol/L, or the use of lipid-lowering medications. Chronic kidney disease was defined as estimated glomerular filtration rate < 60 ml/min/1.73m^2^

*ACEI* angiotensin-converting enzyme inhibitor, *AFABP* adipocyte fatty acid-binding protein, *ARB* angiotensin II receptor blocker, *BMI* body mass index, *CCB* calcium channel blockers, *CKD* chronic kidney disease, *DBP* diastolic blood pressure, *eGFR* estimated glomerular filtration rate, *HbA1c* glycated haemoglobin, *HDL-C* high-density lipoprotein cholesterol, *LDL-C* low-density lipoprotein cholesterol, *SBP* systolic blood pressure

^a^Log-transformed before analysis

^¶^p < 0.05 between quartile 1 and quartile 2

^§^p < 0.05 between quartile 1 and quartile 3

^†^p < 0.05 between quartile 1 and quartile 4

^*^P < 0.05 between quartile 2 and quartile 3

^#^p < 0.05 between quartile 2 and quartile 4

^&^p < 0.05 between quartile 3 and quartile 4

At the time of follow-up echocardiography in these 176 participants, their blood pressure significantly improved, while their eGFR, LDL-C and TG levels decreased compared to baseline. Moreover, there was an increase in the use of gliptins, angiotensin-converting enzyme inhibitors (ACEI) or angiotensin II receptor blockers (ARB) and statins at follow-up. However, their BMI, HbA1c, fasting glucose, HDL-C and the use of insulin, metformin and sulfonylureas were comparable between baseline and at follow-up. (Additional file 1: Table S2).

### Association between AFABP concentration and changes of echocardiography parameters

Compared to baseline, all participants showed a significant increase in IVSd, LVPWd and LV mass at follow-up (all P < 0.01). Moreover, both their LV systolic and diastolic functions deteriorated, as reflected by the decrease in their LVEF, E/A ratio, e′ septal, e′ lateral and increase in their average E/e′ at follow-up (all P < 0.01) (Additional file 1: Table S2).

Table 2 summarizes the relationship between echocardiography parameters and AFABP level of the participants at baseline, follow-up and longitudinal changes. At both time-points, higher AFABP quartiles were associated with increased LV wall thickness (P < 0.01 for IVSd and P < 0.05 for LVPWd) and LV mass (P < 0.01). Similarly, diastolic function was more impaired among those with higher AFABP quartiles, as reflected by their lower e′ septal (P < 0.01), e′ lateral (P < 0.01) and higher average E/e′ (P < 0.05 at baseline and P < 0.01 at follow-up). However, the LV ejection fraction, E/A ratio and LAVi were similar across AFABP quartiles. We further investigated the relationship between their circulating AFABP levels and longitudinal changes of cardiac structure and function. The time between baseline and follow up echocardiography assessments among increased AFABP quartiles was similar [26 months (21–32), 26 months (23–37), 27.5 months (21–35.75) vs. 30.5 months (23.5–37); P = 0.18]. As shown in Table 2, higher AFABP quartiles were associated with a longitudinal increase in LV wall thickness, LV mass and average E/e′ ratio. In particular, participants in the highest AFABP quartile had a significantly greater longitudinal increase in LV mass than those in the lowest AFABP quartile (P < 0.01), as well as a greater increase in average E/e′ ratio compared with those in lower AFABP quartiles (P < 0.01).

**Table 2:** Comparison of echocardiography parameters of study participants stratified by serum AFABP quartiles

	1st Quartile (n = 43)	2nd Quartile (n = 45)	3rd Quartile (n = 44)	4th Quartile (n = 44)	P value
IVSd (mm)					
Baseline	9.84 ± 1.49	10.93 ± 2.02^¶^	10.92 ± 1.98	11.12 ± 1.97^†^	< *0.01*
Follow-up	10.21 ± 1.52	11.83 ± 1.95^¶^	11.31 ± 1.89^§^	12.16 ± 1.86^†^	< *0.01*
Change	0.37 ± 1.15	0.91 ± 1.01	0.39 ± 1.28	1.04 ± 1.28	< *0.05*
LVPWd (mm)					
Baseline	9.29 ± 1.10	8.93 ± 1.16	9.19 ± 1.26	9.69 ± 1.43^#^	< *0.05*
Follow-up	9.04 ± 1.30	9.69 ± 1.49	9.55 ± 1.63	10.02 ± 1.50^†^	< *0.05*
Change	− 0.24 ± 1.32	0.76 ± 1.50^¶^	0.36 ± 1.20	0.34 ± 1.40	< *0.01*
LV mass (g)					
Baseline	137.63 ± 33.74	151.00 ± 43.78	147.46 ± 38.30	168.01 ± 45.08^†^	< *0.01*
Follow-up	139.45 ± 31.84	160.91 ± 43.53	156.20 ± 38.65	180.95 ± 45.57^†&^	< *0.01*
Change	2.24 ± 10.97	9.91 ± 12.10^¶^	8.74 ± 12.23	12.94 ± 16.93^†^	< *0.01*
LVEF (%)					
Baseline	66.02 ± 3.71	65.50 ± 4.16	65.40 ± 4.64	65.61 ± 4.04	0.92
Follow-up	63.81 ± 3.45	64.42 ± 4.46	62.98 ± 5.16	63.64 ± 5.01	0.53
Change	− 2.27 ± 4.31	− 1.24 ± 4.58	− 2.38 ± 6.70	− 1.80 ± 5.14	0.75
E/A					
Baseline	1.05 ± 0.27	0.91 ± 0.24	0.97 ± 0.41	0.90 ± 0.55	0.27
Follow-up	0.93 ± 0.22	0.84 ± 0.18	0.91 ± 0.39	0.78 ± 0.16	< *0.05*
Change	− 0.12 ± 0.24	− 0.07 ± 0.19	− 0.06 ± 0.17	− 0.04 ± 0.21	0.34
e′ septal (cm/s)					
Baseline	8.57 ± 2.11	7.91 ± 2.14	7.36 ± 1.59^§^	6.91 ± 1.99^†^	< *0.01*
Follow-up	7.41 ± 1.80	7.24 ± 1.81	7.30 ± 1.96	6.07 ± 1.72^†#&^	< *0.01*
Change	− 1.15 ± 1.65	− 0.67 ± 2.08	− 0.07 ± 1.67	− 0.79 ± 2.03	0.06
e′ lateral (cm/s)					
Baseline	11.30 ± 2.56	10.65 ± 2.46	10.16 ± 2.41	9.17 ± 2.33^†#^	< *0.01*
Follow-up	10.21 ± 2.47	9.93 ± 2.03	9.18 ± 2.09	8.23 ± 2.48^†#^	< *0.01*
Change	− 1.10 ± 1.77	− 0.74 ± 2.01	− 0.98 ± 1.80	− 0.93 ± 1.87	0.86
Average E/e′					
Baseline	8.45 ± 2.13	8.39 ± 3.25	9.28 ± 2.18	9.80 ± 2.96	< *0.05*
Follow-up	9.12 ± 2.87	8.70 ± 2.35	9.72 ± 2.67	11.66 ± 3.63^†#&^	< *0.01*
Change	0.68 ± 1.65	0.34 ± 2.31	0.44 ± 1.93	1.93 ± 2.25^†#&^	< *0.01*
LAVi (ml/m^2^)					
Baseline	31.86 ± 9.02	29.63 ± 7.04	29.94 ± 9.33	33.21 ± 10.44	0.61
Follow-up	29.10 ± 8.90	28.63 ± 8.74	30.71 ± 9.52	32.26 ± 8.42	0.25
Change	− 2.23 ± 9.03	− 0.34 ± 7.60	0.06 ± 8.68	− 0.68 ± 6.93	0.21

A, trans-mitral late diastolic peak velocity; AFABP, adipocyte fatty acid-binding protein; E, trans-mitral early diastolic peak velocity; e′, early diastolic peak velocity of mitral valve at septal or lateral annulus; IVSd, inter-ventricular septal dimension at end-diastole; LAVi, left atrial volume index; LV, left ventricular; LVEF, LV ejection fraction; LVPWd, LV posterior wall thickness at end-diastole

^¶^ P < 0.05 between 1st quartile and 2nd quartile

^§^ P < 0.05 between 1st quartile and 3rd quartile

^†^ P < 0.05 between 1st quartile and 4th quartile

^#^ P < 0.05 between 2nd quartile and 4th quartile

^&^ P < 0.05 between 3rd quartile and 4th quartile

The association between serum AFABP level and changes of echocardiography parameters was further evaluated by linear regression. In univariate linear regression, AFABP in the highest quartile at baseline was associated with increase in both LV mass and average E/e′ (all P < 0.01) (Additional file 1: Table S3). In multivariable linear regression, baseline AFABP in the highest quartile remained independently associated with both increase in LV mass (P < 0.01) and average E/e′ (P < 0.05), together with hypertension and baseline echocardiography parameters, in a model consisting of age, sex, BMI, smoking status, dyslipidemia and CKD (Table 3).

**Table 3:** Multiple linear regression showing the association of change in left ventricular mass and average E/e′ with serum AFABP quartiles

	ΔLV mass (g)	P value	ΔAverage E/e′	P value
	Standardized β		Standardized β	
Baseline cardiac parameters	− 0.18	< *0.05*	− 0.29	< *0.01*
AFABP quartiles				
1st Quartile	Reference		Reference	
2nd Quartile	0.53	< *0.01*	− 0.28	0.18
3rd Quartile	0.40	0.05	− 0.15	0.48
4th Quartile	0.89	< *0.01*	0.57	< *0.05*
Age (years)	− 0.01	0.87	0.03	0.70
Sex	− 0.14	0.40	− 0.06	0.71
BMI (kg/m^2^)	− 0.01	0.96	− 0.04	0.59
Smoker	0.11	0.55	− 0.02	0.89
Hypertension	0.56	< *0.01*	0.47	< *0.01*
Dyslipidemia	0.13	0.41	0.32	< *0.05*
CKD	− 0.46	< *0.05*	− 0.02	0.93

Baseline cardiac parameters indicate baseline LVM (for change in LVM) and baseline average E/e′ (for change in average E/e′), respectively. Hypertension was defined as BP ≥ 140/90 mmHg or the use of anti-hypertensive medications. Dyslipidemia was defined as fasting triglyceride ≥ 1.69 mmol/L, high-density lipoprotein cholesterol < 1.04 mmol/L in men and < 1.29 mmol/L in women, low- density lipoprotein cholesterol ≥ 2.6 mmol/L, or the use of lipid-lowering medications. Chronic kidney disease was defined as estimated glomerular filtration rate < 60 mL/min/1.73 m^2^

AFABP, adipocyte fatty acid-binding protein; BMI, body mass index; CKD, chronic kidney disease; E, trans-mitral early diastolic peak velocity; e′, early diastolic peak velocity of mitral valve at septal or lateral annulus; LV, left ventricular

### Association between AFABP and MACE

**Fig. 1:**
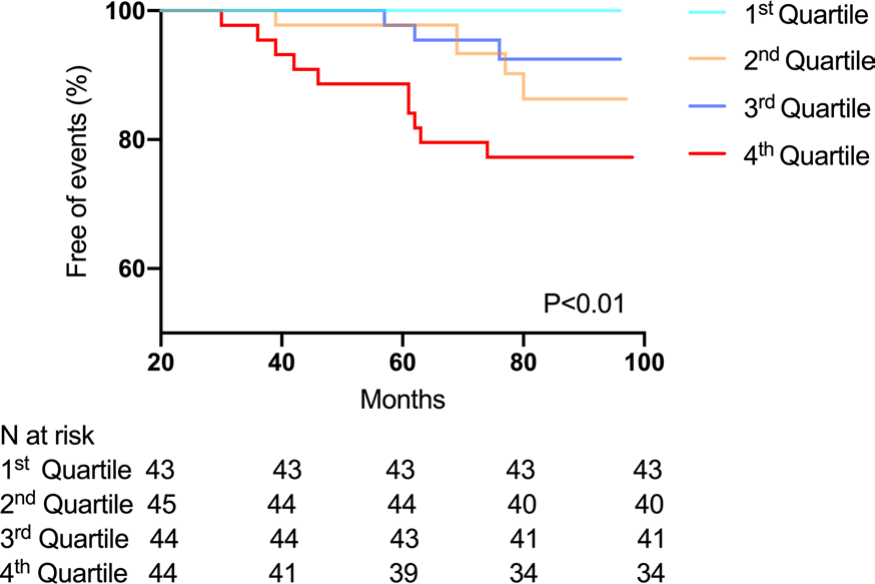
Kaplan–Meier survival curve for incident major adverse cardiovascular events according to quartiles of serum AFABP levels

Among the 176 participants at baseline, 18 of them developed MACE over a median follow-up of 80 months (range 30–98 months), which included 9 cardiovascular deaths, 3 hospitalizations for heart failure, 5 myocardial infarction and 1 stroke. The incidence of MACE was highest among participants with AFABP in the highest quartile (22.7%). Kaplan–Meier survival curve showed that participants with their AFABP in the highest quartile had the highest risk of developing MACE compared to those in the lower AFABP quartiles (Fig. 1, P < 0.01). The clinical characteristics of participants with and without MACE are shown in Additional file 1: Table S4. In multivariable Cox regression analysis, baseline AFABP level was independently associated with the development of MACE (hazard ratio HR 2.65, 1.16–6.05, P < 0.05) in a model adjusted for age, sex, BMI, HbA1c, hypertension, dyslipidemia and CKD at baseline (Table 4). However, the association became attenuated (HR 2.77, 0.99–7.76, P = 0.05) after further adjustments for use of insulin and metformin.

**Table 4:** Multivariable Cox regression analysis showing the association between circulating AFABP level and incident MACE

Model	Adjusted HR for AFABP^a^ (95% CI)	P value
Model 1	4.25 (2.16–8.37)	< *0.01*
Model 2	2.65 (1.16–6.05)	< *0.05*
Model 3	2.77 (0.99–7.76)	0.05

Model 1: include age, sex and body mass index at baseline

Model 2: include all variables in model 1 + hypertension, dyslipidemia, chronic kidney disease and glycated haemoglobin at baseline

Model 3: include all variables in model 2 + use of insulin and metformin at baseline

Hypertension was defined as BP ≥ 140/90 mmHg or the use of anti-hypertensive medications. Dyslipidemia was defined as fasting triglyceride ≥ 1.69 mmol/L, high-density lipoprotein cholesterol < 1.04 mmol/L in men and < 1.29 mmol/L in women, low-density lipoprotein cholesterol ≥ 2.6 mmol/L, or the use of lipid-lowering medications. Chronic kidney disease was defined as estimated glomerular filtration rate < 60 mL/min/1.73 m^2^

AFABP, adipocyte fatty acid-binding protein; CI, confidence interval; HR, hazard ratio; MACE, major adverse cardiovascular events

^a^Log-transformed before analysis

## Discussion

Our study provides the first prospective evaluation of the relationship between circulating AFABP level and longitudinal changes in LV remodelling and diastolic function in patients with T2DM. The key finding of this study was that circulating AFABP level was not only associated with LV remodelling and diastolic dysfunction at baseline but also the longitudinal increase in LV mass and E/e′ ratio after a follow-up of 28 months. Moreover, we demonstrated that circulating AFABP level was an independent predictor of MACE in T2DM patients without CVD at baseline.

Although previous studies have examined the association between circulating AFABP level with LV remodelling and diastolic dysfunction in various groups of patients [19–21, 33], most were of cross-sectional study design and showed conflicting results. Moreover, none has been conducted in exclusively T2DM patients in whom LV hypertrophy and diastolic dysfunction are highly prevalent. Our findings that circulating AFABP level positively correlated with baseline LV mass in T2DM patients concurred with some of the prior studies performed in obese individuals [21] and patients with obstructive sleep apnea [33]. Although some other studies have shown an inverse relationship between circulating AFABP level and LV mass [22, 23], it was recently suggested that the association between LV mass and AFABP level followed a slightly U-shaped curve [23]. In that study, the inverse relation between AFABP and LV mass was most pronounced in AFABP levels below their sex-specific median (18.6 ng/mL in men and 30.69 ng/mL in women), while LV mass began to increase when AFABP was at high levels especially in women. Therefore, the high circulating AFABP levels especially in the highest quartile of our participants (> 29.24 ng/mL in men and > 52.60 ng/mL in women) probably accounted for the positive association between LV mass and AFABP levels observed in our study. Indeed, preclinical studies have shown that the negative inotropic effect of AFABP in rat cardiomyocytes was dose-dependent [34], and that AFABP was associated with cardiac hypertrophy only in the presence of aortic constriction [35]. Therefore, it might be possible that AFABP has different LV remodelling effects depending on concomitant conditions. On the contrary, the relationship between AFABP and diastolic dysfunction is rather consistent. Our findings that baseline serum AFABP level was independently associated with average E/e′, a marker of LV filling pressure [36] and diastolic dysfunction, was in keeping with those reported in the general population [19] and morbidly obese individuals [20].

A major strength of our study is the longitudinal echocardiography assessments using a prospective cohort of T2DM patients without CVD. Echocardiography is a readily available imaging modality that enables the detection of adverse LV remodelling and diastolic dysfunction in T2DM patients [37]. Previous cross-sectional studies using echocardiography have demonstrated that oxidative stress [38], autonomic dysfunction [39], microvascular disease [40], obesity [41] and poor glycemic control [42] were important factors associated with LV hypertrophy and diastolic dysfunction in T2DM patients. In prospective echocardiography studies, retinopathy [43], BNP [44], obesity [45] and female sex [46] were found to be associated with longitudinal changes of LV remodelling and myocardial dysfunction in T2DM patients. In this study, we demonstrated that baseline circulating AFABP level was also independently associated with increase in LV mass and worsening of average E/e′ ratio over a 28-month follow-up period, which provided firm evidence that an elevated circulating AFABP level not only correlated with cross-sectional assessments of LV mass and average E/e′ ratio, but could also predict a further deterioration of LV remodelling and diastolic function in T2DM patients. Our findings therefore demonstrated that circulating AFABP is associated with the development of diabetic cardiomyopathy, which is characterized by the presence of LV hypertrophy and diastolic dysfunction. Although the underlying mechanism of adverse LV remodelling and diastolic dysfunction in T2DM patients is complex and likely to be multifactorial, it has been proposed that circulating AFABP level reflects the accumulation of myocardial neutral lipid in T2DM patients measured by proton magnetic resonance spectroscopy and cardiac triglyceride content in obese mice assessed by lipid chromatography/mass spectrometry [47]. Notably, increased myocardial steatosis has been demonstrated to cause diastolic dysfunction in T2DM patients [48]. Moreover, AFABP has been shown in mice studies to be involved in diabetes-induced cardiac injury through the enhancement of oxidative stress [49]. Collectively, these data support our observation that circulating AFABP was associated with the development and progression of diastolic dysfunction in T2DM. Nonetheless, further studies are required to investigate the underlying pathogenetic mechanism relating AFABP to the adverse LV remodelling and diastolic dysfunction in T2DM patients.

In addition, while previous studies have demonstrated that AFABP predicted MACE in patients with CAD [13], our study extended these findings by showing that high circulating AFABP level was also independently associated with MACE in T2DM patients even without CVD at baseline. Indeed, circulating AFABP level has been associated with adverse cardiovascular events such as stroke [50] and cardiovascular mortality [15, 16] in T2DM patients. Accordingly, our findings that AFABP modulated LV remodelling and worsening of diastolic dysfunction could have provided another explanation to the prognostic role of AFABP in T2DM patients.

Although we [45] and others [51, 52] have previously demonstrated the close relationship between obesity as measured by BMI and LV remodelling, and that an increase in BMI was negatively associated with diastolic function [4, 51, 53], there are also limitations in using BMI to distinguish between fat mass and fat-free mass. In this regard, circulating AFABP level has been shown to correlate with fat mass as measured by bioelectrical impedance and magnetic resonance imaging independent of BMI levels [54]. This notion would thus explain our current observations that AFABP, rather than BMI, correlated more closely to LV remodelling and diastolic dysfunction.

### Clinical implications

It has been increasingly recognized that the presence of LV hypertrophy and diastolic dysfunction is common in patients with T2DM and is associated with adverse clinical outcomes. Nonetheless, the underlying mechanisms of adverse LV remodelling and myocardial dysfunction is uncertain and likely to be multifactorial. Our findings provide clinical evidence that an elevated circulating AFABP level is associated with the development of adverse LV remodelling, diastolic dysfunction and MACE in T2DM. It is therefore of great interest that targeting AFABP might be a potential strategy to prevent adverse LV remodelling, diastolic dysfunction and subsequent adverse events in patients with T2DM. Indeed, prior studies have reported that lifestyle modification with exercise [55, 56], omega-3 fatty acids [57], ARB [58], atorvastatin [59] and sitagliptin [60] may reduce circulating AFABP levels. Moreover, pharmacological inhibition of AFABP via oral inhibitors or neutralizing antibodies has also been demonstrated to alleviate atherosclerosis in animal studies [61]. Whether these agents also reduce the progression of diabetic cardiomyopathy, however, requires confirmation with further studies.

### Limitations

Our study has several limitations. First, the sample size of this prospective study was underpower to estimate the correlation of AFABP with LV remodelling and myocardial dysfunction between BMI subgroups or sex-specific analyses. Second, advanced analysis method such as two-dimension speckle tracking or stress echocardiography could be applied to further confirm the association between AFABP and LV systolic dysfunction. Third, our study only included T2DM patients without existing CVD and future study is required to clarify the role of AFABP in cardiac remodelling and function in T2DM patients with concomitant CVD. Moreover, since only baseline circulating AFABP level was measured, the present study could not evaluate the association of longitudinal changes of circulating AFABP level with LV remodelling and myocardial dysfunction. Lastly, although sodium-glucose co-transporter 2 inhibitors (SGLT2i) has been associated with both changes in AFABP levels and reduction in risk of heart failure in T2DM patients, all of our participants were recruited before SGLT2i became available and hence the effects of SGLT2i in the relationship between AFABP and cardiac remodelling could not be evaluated.

## Conclusion

This prospective study demonstrated that baseline circulating AFABP level was independently associated with a longitudinal increase in LV mass, worsening of diastolic dysfunction and incident MACE among T2DM patients without coexisting CVD. Our findings provided important insights for the planning of future randomized trials that explore targeting AFABP as a potential therapeutic strategy to reduce LV remodelling, diastolic functional deterioration and subsequent adverse events in patients with T2DM.

## Supplementary information

**Additional file 1. Table S1.** Intraobserver and interobserver reproducibility of echocardiography parameters. **Table S2.** Clinical characteristics and echocardiography parameters of participants at baseline and follow-up. **Table S3.** Univariate linear regression showing the variables associated with changes in echocardiography parameters. **Table S4.** Clinical characteristics of participants stratified by incident MACE.

## Data Availability

The datasets generated and/or analysed during the current study are available from the corresponding author on reasonable request.

## Abbreviations

A: Peak transmitral flow velocity in late diastole
ACEI: Angiotensin-converting enzyme inhibitors
AFABP: Adipocyte fatty acid-binding protein
ARB: Angiotensin II receptor blockers
BMI: Body mass index
CAD: Coronary artery disease
CVD: Cardiovascular diseases
eGFR: Estimated glomerular filtration rate
e′: Peak velocity of septal and lateral mitral annulus in early diastole
E: Peak transmitral flow velocity in early diastole
HbA1c: Glycated haemoglobin
HDL-C: High-density lipoprotein cholesterol
IVSd: Inter-ventricular septal dimension at end-diastole
LAVi: Left atrial volume index
LDL-C: Low-density lipoprotein cholesterol
LV: Left ventricular
LVPWd: LV posterior wall thickness at end-diastole
LVEF: LV ejection fraction
MACE: Major adverse cardiovascular events
NT-proBNP: N-terminal pro-brain natriuretic peptide
SCr: Serum creatinine
T2DM: Type 2 diabetes mellitus
TG: Triglyceride
