# Supplementary material for: Association between adipocyte fatty acid-binding protein with left ventricular remodelling and diastolic function in type 2 diabetes: a prospective echocardiography study
Source: Cardiovasc Diabetol. 2020 Nov 24;19:197. doi: 10.1186/s12933-020-01167-5 (PMC7687743; doi:10.1186/s12933-020-01167-5)
Supplement: Supplementary file 1 — Additional file 1. Table S1. Intraobserver and interobserver reproducibility of echocardiography parameters. Table S2. Clinical characteristics and echocardiography parameters of participants at baseline and follow-up. Table S3. Univariate linear regression showing the variables associated with changes in echocardiography parameters. Table S4. Clinical characteristics of participants stratified by incident MACE. [file 12933_2020_1167_MOESM1_ESM.docx]

Additional file 1: Table S1. Intraobserver and interobserver reproducibility of echocardiography parameters

|  | Intraobserver |  |  |  | Interobserver |  |  |
| --- | --- | --- | --- | --- | --- | --- | --- |
|  | Bias (95% LOA) | ICC | P value |  | Bias (95% LOA) | ICC | P value |
| IVSd | 0.04 (-1.54 to 1.62) | 0.92 | 0.81 |  | -0.24 (-2.11 to 1.63) | 0.84 | 0.27 |
| LVPWd | -0.14 (-2.01 to 1.72) | 0.84 | 0.51 |  | -0.35 (-2.11 to 1.40) | 0.84 | 0.10 |
| LV mass | 0.32 (-22.33 to 22.97) | 0.97 | 0.90 |  | -5.18 (-32.99 to 22.64) | 0.94 | 0.12 |
| LVEF | -0.25 (-4.37 to 3.87) | 0.88 | 0.60 |  | 0.65 (-4.41 to 5.71) | 0.81 | 0.27 |
| E/A | 0.02 (-0.12 to 0.16) | 0.97 | 0.30 |  | -0.01 (-0.24 to 0.21) | 0.89 | 0.60 |
| Average E/e’ | 0.09 (-0.96 to 1.14) | 0.98 | 0.49 |  | -0.31 (-1.82 to 1.19) | 0.95 | 0.10 |
| LAVi | -1.23 (-8.48 to 6.03) | 0.92 | 0.16 |  | -1.48 (-12.82 to 9.87) | 0.82 | 0.27 |

Abbreviations: A, trans-mitral late diastolic peak velocity; e’, early diastolic peak velocity of mitral valve at septal or lateral annulus; E, trans-mitral early diastolic peak velocity; ICC, intraclass correlation coefficients; IVSd, inter-ventricular septal dimension at end-diastole; LAVi, left atrial volume index; LOA, limits of agreement; LV, left ventricular; LVEF, LV ejection fraction; LVPWd, LV posterior wall thickness at end-diastole.

Additional file 1: Table S2. Clinical characteristics and echocardiography parameters of participants at baseline and follow-up

|  | Baseline | Follow-up | P value |
| --- | --- | --- | --- |
| BMI (kg/m^2^) | 26±5 | 26±4 | 0.08 |
| SBP (mmHg) | 137±18 | 130±18 | **<0.01** |
| DBP (mmHg) | 80±9 | 75±10 | **<0.01** |
| HbA1c (%) | 7.66±1.25 | 7.58±1.16 | 0.31 |
| Fasting glucose (mmol/L) | 8.29±2.87 | 8.15±2.34 | 0.56 |
| eGFR^a^ (ml/min/1.73m^2^) | 88.69 (73.35-98.23) | 83.98 (65.05-95.99) | **<0.01** |
| Total cholesterol (mmol/L) | 4.31±0.81 | 4.05±0.73 | **<0.01** |
| HDL-C (mmol/L) | 1.30±0.35 | 1.31±0.35 | 0.67 |
| LDL-C (mmol/L) | 2.38±0.63 | 2.15±0.66 | **<0.01** |
| Triglyceride^a^ (mmol/L) | 1.20 (0.80-1.70) | 1.15 (0.80-1.60) | **<0.05** |
| Insulin, n (%) | 79 (44.9) | 79 (44.9) | 1.00 |
| Metformin, n (%) | 166 (94.3) | 164 (93.2) | 0.69 |
| Sulfonylureas, n (%) | 93 (52.8) | 85 (48.3) | 0.06 |
| Gliptins, n (%) | 36 (20.5) | 61 (34.7) | **<0.01** |
| ACEI/ARB, n (%) | 107 (60.8) | 118 (67.0) | **<0.05** |
| β-blocker, n (%) | 60 (34.1) | 60 (34.1) | 1.00 |
| CCB, n (%) | 83 (47.2) | 90 (51.1) | 0.09 |
| Diuretics, n (%) | 16 (9.1) | 18 (10.2) | 0.75 |
| Statin, n (%) | 94 (53.4) | 111 (63.1) | **<0.01** |
| IVSd (mm) | 10.71±1.93 | 11.38±1.95 | **<0.01** |
| LVPWd (mm) | 9.27±1.26 | 9.58±1.51 | **<0.01** |
| LV mass (g) | 151.08±41.64 | 159.59±42.58 | **<0.01** |
| LVEF (%) | 65.61±4.11 | 63.69±4.58 | **<0.01** |
| E/A | 0.94±0.30 | 0.87±0.26 | **<0.01** |
| e’ septal (cm/s) | 7.68±2.05 | 7.02±1.89 | **<0.01** |
| e’ lateral (cm/s) | 10.31±2.54 | 9.37±2.40 | **<0.01** |
| Average E/e’ | 8.96±2.70 | 9.79±3.14 | **<0.01** |
| LAVi (ml/m^2^) | 31.16±9.09 | 30.37±8.89 | 0.22 |

Abbreviations: A, trans-mitral late diastolic peak velocity; ACEI, angiotensin-converting enzyme inhibitor; ARB, angiotensin II receptor blocker; BMI, body mass index; CCB, calcium channel blockers; DBP, diastolic blood pressure; E, trans-mitral early diastolic peak velocity; e’, early diastolic peak velocity of mitral valve at septal or lateral annulus; eGFR, estimated glomerular filtration rate; HbA1c, glycated haemoglobin; HDL-C, High-density lipoprotein cholesterol; IVSd, inter-ventricular septal dimension at end-diastole; LAVi, left atrial volume index; LDL-C, low-density lipoprotein cholesterol; LV, left ventricular; LVEF, LV ejection fraction; LVPWd, LV posterior wall thickness at end-diastole; SBP, systolic blood pressure.

^a^ Log-transformed before analysis

Additional file 1: Table S3. Univariate linear regression showing the variables associated with changes in echocardiography parameters

|  | △LV mass (g) |  |  | △LVEF (%) |  |  | △E/A |  |  | △Average E/e’ |  |  | △LAVi (ml/m^2^) |  |
| --- | --- | --- | --- | --- | --- | --- | --- | --- | --- | --- | --- | --- | --- | --- |
|  | β | P value |  | β | P value |  | β | P value |  | β | P value |  | β | P value |
| Baseline cardiac parameters | -0.03 | 0.21 |  | -0.70 | **<0.01** |  | -0.36 | **<0.01** |  | -0.13 | **<0.05** |  | -0.42 | **<0.01** |
| AFABP quartiles |  |  |  |  |  |  |  |  |  |  |  |  |  |  |
| 1^st^ Quartile | Reference |  |  | Reference |  |  | Reference |  |  | Reference |  |  | Reference |  |
| 2^nd^ Quartile | 7.67 | **<0.01** |  | 1.03 | 0.37 |  | 0.05 | 0.26 |  | -0.34 | 0.45 |  | 1.89 | 0.30 |
| 3^rd^ Quartile | 6.50 | **<0.05** |  | -0.11 | 0.92 |  | 0.06 | 0.17 |  | -0.24 | 0.59 |  | 2.30 | 0.20 |
| 4^th^ Quartile | 10.71 | **<0.01** |  | 0.47 | 0.69 |  | 0.08 | 0.08 |  | 1.25 | **<0.01** |  | 1.56 | 0.39 |
| Age (years) | 0.09 | 0.39 |  | 0.04 | 0.43 |  | 0.00 | 0.29 |  | 0.01 | 0.66 |  | 0.16 | **<0.05** |
| Sex | -3.01 | 0.15 |  | 2.18 | **<0.01** |  | -0.05 | 0.08 |  | 0.07 | 0.83 |  | -0.22 | 0.86 |
| Smoker | 0.49 | 0.83 |  | -0.08 | 0.93 |  | -0.09 | **<0.05** |  | 0.07 | 0.84 |  | -0.09 | 0.95 |
| BMI (kg/m^2^) | 0.26 | 0.25 |  | -0.22 | **<0.05** |  | 0.00 | 0.90 |  | 0.04 | 0.31 |  | 0.07 | 0.61 |
| Hypertension | 8.56 | **<0.01** |  | 1.53 | 0.11 |  | 0.03 | 0.47 |  | 0.90 | **<0.05** |  | 1.37 | 0.36 |
| Dyslipidemia | 2.81 | 0.21 |  | 0.25 | 0.78 |  | 0.03 | 0.39 |  | 0.87 | **<0.05** |  | 1.64 | 0.24 |
| CKD | -1.20 | 0.69 |  | -1.12 | 0.36 |  | 0.02 | 0.62 |  | 0.54 | 0.27 |  | 3.06 | 0.09 |
| HbA1c (%) | -1.30 | 0.11 |  | 0.05 | 0.87 |  | 0.03 | **<0.05** |  | 0.17 | 0.19 |  | 1.47 | **<0.01** |
| Total cholesterol (mmol/L) | -0.41 | 0.75 |  | -0.75 | 0.14 |  | -0.01 | 0.67 |  | -0.58 | **<0.01** |  | 0.22 | 0.77 |
| HDL-C (mmol/L) | 0.45 | 0.88 |  | 1.26 | 0.28 |  | -0.04 | 0.42 |  | -0.80 | 0.10 |  | -0.18 | 0.92 |
| LDL-C (mmol/L) | 0.29 | 0.86 |  | -0.67 | 0.30 |  | -0.02 | 0.43 |  | -0.76 | **<0.01** |  | -0.43 | 0.67 |
| Triglyceride^a^ (mmol/L) | -2.33 | 0.19 |  | -0.34 | 0.63 |  | 0.02 | 0.43 |  | 0.21 | 0.47 |  | 0.83 | 0.45 |
| Insulin | -2.00 | 0.34 |  | -1.60 | **0.05** |  | 0.05 | 0.14 |  | 0.61 | 0.06 |  | 1.89 | 0.14 |
| ACEI/ARB | 5.66 | **<0.01** |  | 0.95 | 0.26 |  | 0.00 | 0.94 |  | 0.31 | 0.35 |  | 1.09 | 0.41 |
| CCB | 6.81 | **<0.01** |  | 0.04 | 0.96 |  | 0.03 | 0.39 |  | 0.74 | **<0.05** |  | 2.58 | **<0.05** |
| β-blocker | 3.56 | 0.11 |  | 1.52 | 0.08 |  | 0.01 | 0.71 |  | 0.56 | 0.11 |  | 1.29 | 0.34 |
| Diuretics | 0.03 | 1.00 |  | 0.93 | 0.53 |  | 0.11 | 0.06 |  | 0.85 | 0.15 |  | 1.54 | 0.50 |
| Statin | 3.27 | 0.12 |  | 0.72 | 0.38 |  | 0.03 | 0.39 |  | 0.95 | **<0.01** |  | 0.25 | 0.85 |

Baseline cardiac parameters indicate baseline LVM (for change in LVM), baseline LVEF (for change in LVEF), baseline E/A (for change in E/A), baseline average E/e’ (for change in average E/e’) and baseline LAVi (for change in LAVi), respectively. Hypertension was defined as BP ≥140/90mmHg or the use of anti-hypertensive medications. Dyslipidemia was defined as fasting triglyceride ≥1.69 mmol/L, high-density lipoprotein cholesterol <1.04 mmol/L in men and <1.29 mmol/L in women, low-density lipoprotein cholesterol ≥2.6 mmol/L, or the use of lipid-lowering medications. Chronic kidney disease was defined as estimated glomerular filtration rate <60 ml/min/1.73m^2^.

Abbreviations: A, trans-mitral late diastolic peak velocity; ACEI, angiotensin-converting enzyme inhibitor; AFABP, adipocyte fatty acid-binding protein; ARB, angiotensin II receptor blocker; BMI, body mass index; CCB, calcium channel blockers; CKD, chronic kidney disease; E, trans-mitral early diastolic peak velocity; e’, early diastolic peak velocity of mitral valve at septal or lateral annulus; HbA1c, glycated haemoglobin; HDL-C, High-density lipoprotein cholesterol; LAVi, left atrial volume index; LDL-C, Low-density lipoprotein cholesterol; LV, left ventricular; LVEF, LV ejection fraction.

^a^ Log-transformed before analysis

Additional file 1: Table S4. Clinical characteristics of participants stratified by incident MACE

|  | Incident MACE (n=18) | Without incident MACE (n=158) | HR (95%CI) | Unadjusted P value |
| --- | --- | --- | --- | --- |
| AFABP^a^ (ng/mL) | 49.69 (21.90-93.21) | 23.60 (15.88-33.82) | 4.03 (2.20-7.40) | **<0.01** |
| Age (years) | 65±10 | 60±10 | 1.04 (1.00-1.09) | 0.08 |
| Men, n (%) | 11 (61.1) | 83 (52.5) | 1.40 (0.54-3.61) | 0.49 |
| Diabetes duration (years) | 19±7 | 16±7 | 1.04 (0.98-1.10) | 0.26 |
| BMI (kg/m^2^) | 28±6 | 26±4 | 1.08 (1.00-1.16) | **0.05** |
| SBP (mmHg) | 142±17 | 136±19 | 1.01 (0.99-1.04) | 0.23 |
| DBP (mmHg) | 78±9 | 80±9 | 0.97 (0.92-1.02) | 0.28 |
| Smoker, n (%) | 7 (38.9) | 40 (25.3) | 1.80 (0.70-4.65) | 0.22 |
| Hypertension, n (%) | 17 (94.4) | 116 (73.4) | 5.73 (0.76-43.07) | 0.09 |
| Dyslipidemia, n (%) | 17 (94.4) | 104 (65.8) | 8.54 (1.14-64.23) | **<0.05** |
| CKD, n (%) | 8 (44.4) | 17 (10.8) | 5.38 (2.12-13.64) | **<0.01** |
| HbA1c (%) | 8.45±1.70 | 7.59±1.18 | 1.44 (1.11-1.87) | **<0.01** |
| Fasting glucose (mmol/L) | 8.46±3.81 | 8.13±2.60 | 1.04 (0.90-1.22) | 0.63 |
| eGFR^a^ (mL/min/1.73m^2^) | 63.55 (42.13-89.73) | 89.63 (77.29-98.46) | 0.06 (0.02-0.16) | **<0.01** |
| Total cholesterol (mmol/L) | 4.38±0.65 | 4.29±0.82 | 1.12 (0.63-2.01) | 0.69 |
| HDL-C (mmol/L) | 1.20±0.30 | 1.32±0.36 | 0.48 (0.11-2.09) | 0.33 |
| LDL-C (mmol/L) | 1.94±0.53 | 2.18±0.67 | 0.91 (0.43-1.92) | 0.81 |
| Triglyceride^a^ (mmol/L) | 1.60 (1.15-2.40) | 1.10 (0.80-1.60) | 2.45 (1.25-4.79) | **<0.01** |
| Insulin, n (%) | 14 (77.8) | 65 (41.1) | 4.73 (1.56-14.40) | **<0.01** |
| Metformin, n (%) | 14 (77.8) | 152 (96.2) | 0.20 (0.07-0.60) | **<0.01** |
| Sulfonylureas, n (%) | 5 (27.8) | 88 (55.7) | 0.35 (0.12-1.01) | **0.05** |
| Gliptins, n (%) | 2 (11.1) | 34 (21.5) | 0.64 (0.14-2.88) | 0.56 |
| ACEI/ARB, n (%) | 14 (77.8) | 93 (58.9) | 2.36 (0.78-7.17) | 0.13 |
| β-blocker, n (%) | 10 (55.6) | 50 (31.6) | 2.59 (1.02-6.57) | **<0.05** |
| CCB, n (%) | 12 (66.7) | 71 (44.9) | 2.32 (0.87-6.20) | 0.09 |
| Diuretics, n (%) | 5 (27.8) | 11 (7.0) | 4.84 (1.72-13.60) | **<0.01** |
| Statin, n (%) | 15 (83.3) | 79 (50.0) | 4.77 (1.38-16.47) | **<0.05** |

Hypertension was defined as BP ≥140/90mmHg or the use of anti-hypertensive medications. Dyslipidemia was defined as fasting triglyceride ≥1.69 mmol/L, high-density lipoprotein cholesterol <1.04 mmol/L in men and <1.29 mmol/L in women, low-density lipoprotein cholesterol ≥2.6 mmol/L, or the use of lipid-lowering medications. Chronic kidney disease was defined as estimated glomerular filtration rate <60 ml/min/1.73m^2^.

Abbreviations: ACEI, angiotensin-converting enzyme inhibitor; AFABP, adipocyte fatty acid-binding protein; ARB, angiotensin II receptor blocker; BMI, body mass index; CCB, calcium channel blockers; CI, confidence interval; CKD, chronic kidney disease; DBP, diastolic blood pressure; eGFR, estimated glomerular filtration rate; HbA1c, glycated haemoglobin; HDL-C, High-density lipoprotein cholesterol; HR, hazard ratio; LDL-C, low-density lipoprotein cholesterol; MACE, major adverse cardiovascular events; SBP, systolic blood pressure.

^a^ Log-transformed before analysis
